# Stage-dependent microbial succession and metabolite profiles of *Phyllanthus emblica* L. fruit wines produced under different temperature and pomace treatments

**DOI:** 10.1016/j.fochx.2026.104501

**Published:** 2026-09-23

**Authors:** Tingmei Qi, Xinyong Zhang, Ni Liu, Mingju Wan, Yang Tian, Qi Lin, Yuanyuan Bao

**Affiliations:** a College of Food Science and Technology, Yunnan Agricultural University, Kunming 650201, China; b College of Agriculture and Biotechnology, Yunnan Agricultural University, Kunming, Yunnan 650201, China; c Engineering Research Center of Development and Utilization of Food and Drug Homologous Resources, Ministry of Education, Yunnan Agricultural University, Kunming 650201, China; d Yunnan Key Laboratory of Precision Nutrition and Personalized Food Manufacturing, Yunnan Agricultural University, Kunming 650201, China

**Keywords:** *Phyllanthus emblica* L., Low-temperature fermentation, Pomace, Microbial succession, Untargeted metabolomics, Volatile profile

## Abstract

Juice wine fermented at 20 °C (FW) and pomace-containing juice wine fermented at 20 °C (PW) were investigated, with juice wine fermented at 26 °C (CK) included as a conventional-temperature reference. Microbial communities and volatile profiles were characterised in all treatments using 16S rRNA gene and ITS amplicon sequencing and HS-SPME–GC–MS, whereas untargeted LC–MS metabolomics compared FW and PW. The treatments exhibited distinct stage-dependent microbial succession and chemical profiles. *Saccharomyces*-assigned sequences dominated the fungal communities, whereas bacterial composition varied among stages and treatments, with a transient increase in *Gluconobacter*-assigned sequences in PW. Overall, 2936 non-volatile features were tentatively annotated, and differential features were mainly associated with organic acids and carbohydrates. Volatile profiles differed in the relative proportions of alcohol-, ester-, acid-, and terpenoid-related features. Microbe–metabolite correlations were stage dependent but did not establish causality. These findings provide a compositional reference for *P. emblica* wine fermentation.

## Highlights

• Fermentation stage and treatment reshaped *P. emblica* L. microbial communities.
• *Saccharomyces* dominated fungi, while *Gluconobacter* transiently enriched in PW.
• Pomace addition altered non-volatile profiles across fermentation stages.
• FW and PW differed in proportional volatile-feature composition at 20 °C.
• Microbe–metabolite associations were stage dependent and exploratory.

## Graphical abstract

**Figure:**
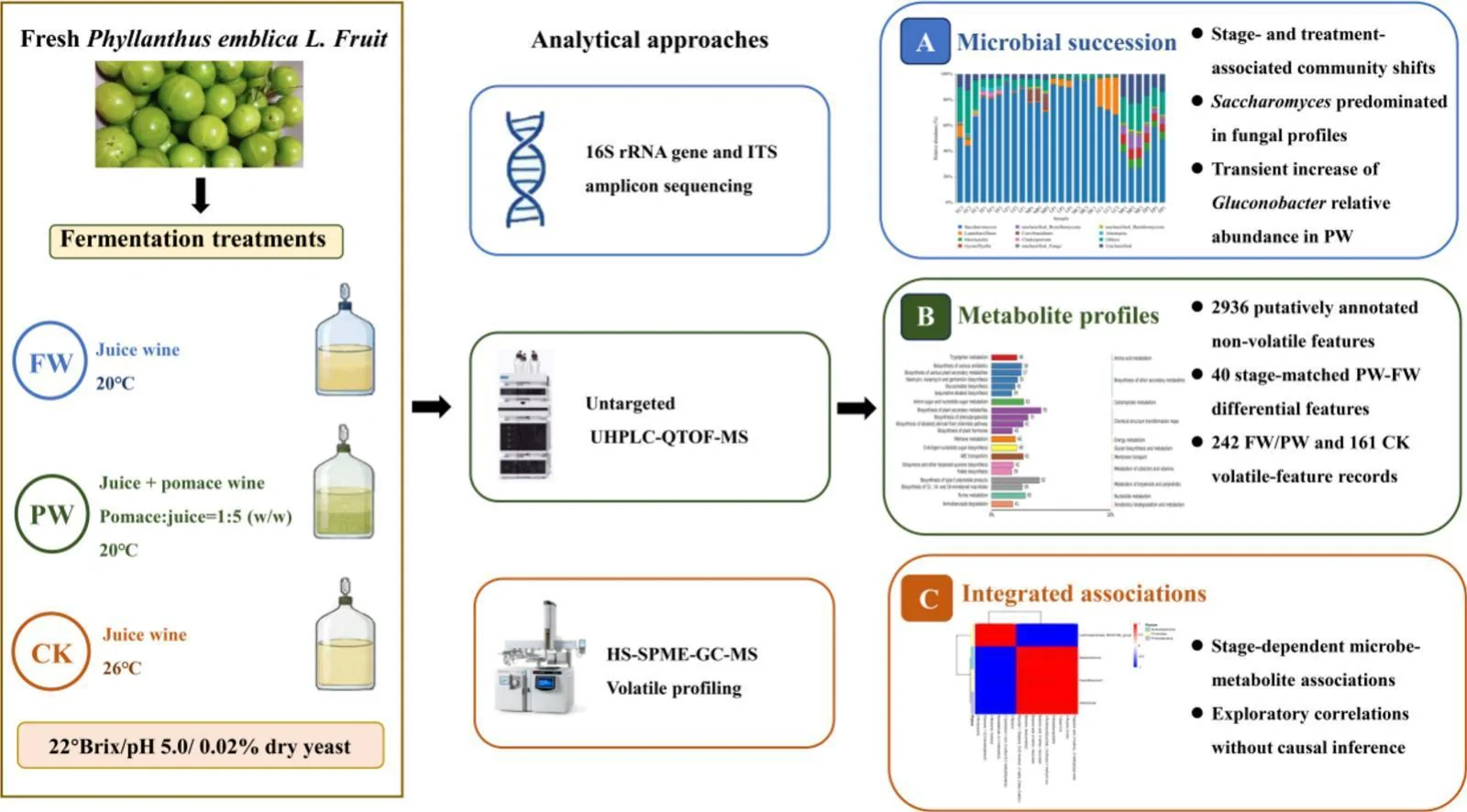

## 1 Introduction

*Phyllanthus emblica* L. (syn. *Emblica officinalis* Gaertn.), commonly known as amla or Indian gooseberry, is an edible fruit widely cultivated and processed in tropical and subtropical Asia. It is rich in ascorbic acid and phenolic constituents, including tannins, phenolic acids, and flavonoids, which contribute to its antioxidant properties (Mahajan et al., 2023). However, these constituents, together with the high organic-acid content of the fruit, are also associated with pronounced sourness, bitterness, and astringency. *P. emblica* additionally produces a characteristic sweet aftertaste attributed to specific taste-active compounds (Che et al., 2022). Although these compositional characteristics support its development into value-added beverages, excessive acidity and astringency may restrict consumer acceptance. Processing and fermentation strategies that moderate these sensory properties while retaining the distinctive composition of the fruit are therefore of interest (Zhang et al., 2024).

Alcoholic fermentation is an effective approach for diversifying fruit-derived products. During fermentation, inoculated *Saccharomyces cerevisiae* converts fermentable sugars into ethanol and produces secondary metabolites that contribute to flavour. Nevertheless, the initial organic-acid and phenolic profiles are determined primarily by the raw-fruit matrix and may subsequently be modified through extraction, oxidation, polymerization, precipitation, esterification, and microbial transformation. Consequently, the final acidity, bitterness, and astringency of fruit wine cannot be attributed to fermentation temperature alone. Temperature can, however, affect yeast growth and carbon- and nitrogen-related metabolism, thereby altering the formation and relative distribution of volatile and non-volatile metabolites. Previous studies have shown that low-temperature fermentation can reshape yeast-derived metabolite profiles and modify the distributions of ethanol, organic acids, esters, higher alcohols, and other flavour-related components. These responses depend on the yeast strain, substrate matrix, and fermentation conditions (Du et al., 2022; Du et al., 2024; González Flores et al., 2024). Low-temperature fermentation should therefore be regarded as a process variable that modulates fermentation metabolism rather than as a treatment that universally improves wine quality.

The chemical profile of fruit wine comprises volatile components contributing to aroma and non-volatile metabolites influencing acidity, sweetness, bitterness, astringency, and mouthfeel. These profiles are affected by fruit cultivar, substrate composition, yeast strain, microbial interactions, fermentation temperature, and processing conditions. Mass-spectrometry-based metabolomics has consequently been increasingly applied to characterize treatment-associated compositional differences in fruit wines. Combined HS-SPME–GC–MS and UHPLC–MS analyses have revealed changes in volatile and non-volatile metabolites during jujube- and plum-wine fermentation (Li et al., 2025; Zhao et al., 2024). Integration of metabolomics with microbial-community analysis can further reveal associations between microbial succession and metabolite variation (de Celis et al., 2024; Wang et al., 2024). However, DNA-based amplicon sequencing cannot distinguish viable and metabolically active microorganisms from inactive cells or residual DNA. Microbial–metabolite correlations should therefore be interpreted as statistical associations rather than direct evidence of microbial production, pathway activation, or metabolic regulation.

Fruit pomace is an underutilized by-product of juice processing that retains dietary fiber, phenolic constituents, organic acids, residual sugars, and other components. *P. emblica* pomace may therefore represent a valuable food ingredient and fermentation substrate (Gan et al., 2022; Muzaffar et al., 2023). Dried *P. emblica* pomace was previously reported to contain approximately 76.60% total dietary fiber on a dry-weight basis, including 64.88% insoluble dietary fiber and 11.72% soluble dietary fiber (Wu et al., 2022). These values are specific to dried pomace and may vary with fruit maturity, juice-extraction and drying conditions, analytical procedures, and reporting basis. Incorporating pomace into fruit-wine fermentation may modify solid–liquid extraction, nutrient availability, phenolic composition, and volatile profiles. Pomace-ratio adjustment has been shown to affect the flavour and sensory characteristics of grape wine, whereas ciders produced from apple juice and corresponding pomace exhibited distinct physicochemical, volatile, and sensory profiles (Shi et al., 2023; Xu et al., 2025). Nevertheless, pomace-associated effects remain dependent on the fruit matrix, pomace proportion, and fermentation conditions.

Our previous study established a low-temperature fermentation process for *P. emblica* fruit wine and evaluated its fermentation kinetics, physicochemical characteristics, antioxidant activity, and sensory properties (Liu et al., 2026). However, stage-dependent microbial succession and its associations with volatile and non-volatile metabolite variation were not investigated. In the present study, juice wine fermented at 20 °C (FW), pomace-containing juice wine fermented at 20 °C (PW), and juice wine fermented at 26 °C (CK) were characterised using bacterial 16S rRNA gene and fungal ITS amplicon sequencing and HS-SPME–GC–MS. Untargeted UHPLC–QTOF–MS metabolomics was used specifically to compare the two low-temperature treatments, FW and PW, whereas CK served as a conventional-temperature juice-wine reference. The objectives were to: (i) characterize stage-dependent microbial-community profiles among FW, PW, and CK; (ii) compare non-volatile metabolite profiles between FW and PW and characterize treatment-associated differences in volatile profiles; and (iii) evaluate statistical associations between microbial taxa and metabolite variation. Because a pomace-containing treatment fermented at 26 °C was not included, FW versus PW was interpreted as a pomace-associated comparison at 20 °C, FW versus CK as a temperature-associated reference within the juice-only matrix, and PW versus CK as an overall treatment comparison involving both temperature and substrate composition.

## 2 Materials and methods

### 2.1 Raw materials and chemicals

Fresh *Phyllanthus emblica* L. fruits were purchased in November 2024 from Shiping County, Honghe Hani and Yi Autonomous Prefecture, Yunnan Province, China. Commercially mature fruits of full size and firm texture, without visible disease, insect damage, or mechanical injury, were selected. The freshly prepared juice contained 6.58 ± 0.07% total sugar and had a total soluble solids content of 8.13 ± 0.02°Brix, an initial pH of 2.94, and a total acidity of 1.76 ± 0.05 g/L, expressed as tartaric acid. A 10 kg portion of fresh fruit was used for each fermentation treatment.

Angel SY active dry wine yeast was obtained from Angel Yeast Co., Ltd. (Yichang, Hubei, China). Food-grade citric acid, sucrose, and sodium chloride were supplied by Shandong Ensign Industry Co., Ltd., COFCO Sugar Holding Co., Ltd., and Jiangsu Salt Industry Group Co., Ltd., respectively. Food-grade sodium carbonate was obtained from Jiahe Xuri Co., Ltd., and food-grade sodium D-isoascorbate was obtained from Zhucheng Huayuan Bioengineering Co., Ltd. (Zhucheng, Shandong, China). All other chemicals were of analytical grade unless otherwise specified.

### 2.2 Preparation of *Phyllanthus emblica* L. fruit wines

The fermentation procedure and endpoint criteria were based primarily on Liu et al. (2026), whereas the conventional fermentation temperature of 26 °C used for CK was selected with reference to Chen et al. (2026).

Fresh *P. emblica* L. fruits were washed and deseeded, and the pulp was immersed in an anti-browning solution containing 0.1% (w/v) citric acid and 1.0% (w/v) sodium chloride. The treated pulp was mixed with potable water at a pulp-to-water ratio of 1:2 (w/v) and pressed. Sodium D-isoascorbate was added to the resulting juice at 0.03% (w/v), after which the juice and freshly separated wet pomace were separated by filtration.

Three treatments were prepared concurrently: juice wine fermented at 20 °C (FW), pomace-containing juice wine fermented at 20 °C (PW), and juice wine fermented at 26 °C as the conventional-temperature control (CK). For PW, freshly separated wet pomace was added on day 0, immediately before yeast inoculation, at a wet-pomace-to-juice ratio of 1:5 (w/w). No pomace was added to FW or CK.

The total soluble solids content of each must was adjusted to 22°Brix using sucrose. The pH was adjusted to 5.0 using food-grade sodium carbonate, with citric acid used for downward correction when necessary. Each must was thoroughly mixed until the added ingredients were completely dissolved.

Angel SY active dry wine yeast was rehydrated in water at a yeast-to-water ratio of 1:9 (w/w), activated at 37 °C for 25 min, and inoculated into each must at 0.02% (w/v), equivalent to 0.20 g/L.

Each must was thoroughly homogenized and dispensed into multiple 250 mL glass fermentation bottles, with 200 mL of must placed in each bottle. Each bottle was fitted with a water-filled airlock. FW and PW were fermented at 20 °C, whereas CK was fermented at 26 °C. Except for fermentation temperature and the addition of pomace to PW, all preparation and fermentation conditions were maintained consistently among the treatments.

Fermentation was considered complete when visible gas evolution had ceased and the alcohol content had stabilized. Sampling and sample storage were performed as described in Section 2.3.1.

### 2.3 Microbial community analysis of *Phyllanthus emblica* L. fruit wines

#### 2.3.1 Sample collection

Sampling times were selected according to the fermentation kinetics established in our previous study (Liu et al., 2026). Samples collected at the start of fermentation on day 0 were defined as the initial fermentation-stage samples and designated EF, EP, and EC for the FW, PW, and CK treatments, respectively. The middle fermentation stage corresponded to the period of active fermentation and the maximum yeast-cell count. Accordingly, FW, PW, and CK samples collected on days 10, 5, and 6 were designated MF, MP, and MC, respectively. The late fermentation stage was characterised by stable total sugar and alcohol contents and the absence of visible gas evolution. FW, PW, and CK samples collected on days 15, 13, and 11 were therefore designated LF, LP, and LC, respectively.

At each predefined sampling stage, three bottles were randomly selected from each treatment and destructively sampled. Each selected bottle was opened and sampled only at its designated stage and was not returned to the fermentation experiment. This design resulted in three bottle-level fermentation replicates for each treatment–stage combination, comprising nine treatment–stage combinations and 27 samples in total.

Each sample was divided into separate aliquots for bacterial and fungal community analyses, untargeted non-volatile metabolomic analysis, and volatile-profile analysis. All 27 samples were included in these analyses. All aliquots were immediately frozen in liquid nitrogen and stored at −80 °C until analysis.

#### 2.3.2 DNA extraction, PCR amplification, and PacBio sequencing

Microbial cells were collected from 2.0 mL of each wine sample by centrifugation at 13,400 ×*g* for 2 min. The supernatant was discarded, and the resulting pellet was retained. When no clearly visible pellet was obtained, approximately 50–100 μL of residual liquid was retained to minimize microbial-cell loss.

The pellet was resuspended in 600 μL of Buffer SNA, followed by the addition of 10 μL of RNase A. Cell disruption was performed using a bead mill at 6 m/s for six cycles, with each cycle consisting of 20 s of homogenization followed by a 20 s interval. After centrifugation at 12,000 rpm for 1 min, 400–500 μL of the supernatant was transferred to a new tube and mixed with 200 μL of HA Removal Buffer CN pre-cooled to 2–8 °C. The mixture was vortexed for 3–5 s and centrifuged at 12,000 rpm for 1 min. Subsequently, 500 μL of the resulting supernatant was subjected to magnetic-bead-based DNA purification using a TGuide S96 Magnetic Soil/Fecal DNA Kit (TIANGEN Biotech (Beijing) Co., Ltd., Beijing, China). DNA was eluted in 60–100 μL of elution buffer.

DNA integrity was evaluated by electrophoresis on a 1.8% agarose gel, and DNA concentration and purity were assessed using a NanoDrop 2000 spectrophotometer (Thermo Fisher Scientific, Waltham, MA, USA).

The nearly full-length bacterial 16S rRNA gene was amplified using primers 27F (5′-AGRGTTTGATYNTGGCTCAG-3′) and 1492R (5′-TASGGHTACCTTGTTASGACTT-3′). The fungal internal transcribed spacer region was amplified using primers ITS1F (5′-CTTGGTCATTTAGAGGAAGTAA-3′) and ITS4 (5′-TCCTCCGCTTATTGATATGC-3′). Sample-specific PacBio barcode sequences were incorporated at the 5′ ends of the primers to enable sample demultiplexing.

PCR amplification was performed using KOD One PCR Master Mix (TOYOBO Co., Ltd., Osaka, Japan) under the following conditions: initial denaturation at 95 °C for 2 min; 25 cycles of denaturation at 98 °C for 10 s, annealing at 55 °C for 30 s, and extension at 72 °C for 90 s; followed by a final extension at 72 °C for 2 min.

PCR products were purified using VAHTS DNA Clean Beads (Vazyme Biotech Co., Ltd., Nanjing, China) and quantified using a Qubit dsDNA High-Sensitivity Assay Kit and a Qubit 3.0 fluorometer (Thermo Fisher Scientific). Equimolar quantities of the purified amplicons were pooled for SMRTbell library construction. After library-quality assessment, qualified libraries were sequenced on a PacBio Sequel II platform by Biomarker Technologies Co., Ltd. (Beijing, China).

#### 2.3.3 Sequence processing and taxonomic annotation

PacBio sequencing data in BAM format were processed using SMRT Link to generate circular consensus sequences (CCS). CCS reads were assigned to individual samples according to their barcode sequences using Lima v1.7.0. Primer sequences were removed, and sequence-length filtering was performed using Cutadapt v1.9.1 to obtain clean CCS reads. Chimeric sequences were identified and removed using UCHIME v4.2, yielding effective CCS reads.

Effective CCS reads were clustered into operational taxonomic units (OTUs) at 97.0% sequence identity using USEARCH v12, and a representative sequence was selected for each OTU. Low-abundance OTUs represented by fewer than two reads across the entire dataset were removed before downstream analysis.

Representative bacterial 16S rRNA gene OTU sequences were taxonomically annotated against the SILVA release 138 database, whereas representative fungal ITS OTU sequences were annotated against the NCBI nucleotide database, using BLAST-based sequence alignment combined with a naïve Bayes classifier. Taxonomic abundance tables at the kingdom, phylum, class, order, family, genus, and species levels were generated using QIIME 2 v2020.6. Sequences classified as unclassified or unidentified at a given taxonomic level were excluded only from the corresponding level-specific taxonomic summary.

Alpha diversity was evaluated using the ACE, Chao1, Shannon, and Simpson indices. Beta diversity was assessed using binary Jaccard and Bray–Curtis distances for both bacterial and fungal communities. Weighted and unweighted UniFrac distances were additionally calculated for the bacterial community. Principal component analysis (PCA) based on OTU relative-abundance profiles was performed in R to visualize differences in bacterial and fungal community composition among samples. Differences in beta diversity among the treatment–stage groups were evaluated using permutational multivariate analysis of variance (PERMANOVA) and analysis of similarities (ANOSIM) implemented in the vegan package in R.

Differentially abundant taxa were identified using linear discriminant analysis effect size (LEfSe), with an LDA score threshold of 4.0. Bioinformatic analyses and data visualisation were performed using QIIME 2 v2020.6, R, and the BMKCloud platform (Biomarker Technologies Co., Ltd., Beijing, China).

The raw bacterial 16S rRNA gene and fungal ITS amplicon sequencing data have been deposited in the NCBI Sequence Read Archive under BioProject accession number PRJNA1503743.

### 2.4 Untargeted non-volatile metabolomic analysis of *Phyllanthus emblica* L. fruit wine

#### 2.4.1 Metabolite extraction

An aliquot of each wine sample (100 μL) was mixed with 500 μL of methanol/acetonitrile (1:1, v/v) containing 2-chloro-*L*-phenylalanine at 20 mg/L in the extraction solvent as an internal reference (Dong et al., 2025; Ren et al., 2023). The mixture was vortexed for 30 s, sonicated in an ice–water bath for 10 min, and maintained at −20 °C for 1 h. After centrifugation at 12,000 rpm for 15 min at 4 °C, 500 μL of the supernatant was transferred to a new tube and evaporated to dryness using a vacuum concentrator.

The dried extract was reconstituted in 160 μL of acetonitrile/water (1:1, v/v), vortexed for 30 s, sonicated in an ice–water bath for 10 min, and centrifuged at 12,000 rpm for 15 min at 4 °C. Subsequently, 120 μL of the supernatant was transferred to a 2 mL LC–MS vial.

The FW and PW samples were analysed together in an 18-sample analytical batch, whereas the CK samples were analysed in a separate analytical batch using the same extraction workflow. For each analytical batch, a pooled quality-control (QC) sample was prepared by combining 10 μL aliquots of all individual extracts included in that batch (Hai et al., 2023; Zhang, Wei, et al., 2025).

#### 2.4.2 UHPLC–QTOF–MS analysis

Metabolic profiling was performed using an ACQUITY UPLC I-Class PLUS system coupled to a Xevo G2-XS QTof high-resolution mass spectrometer (Waters Corporation, Milford, MA, USA). Chromatographic separation was achieved using an ACQUITY UPLC HSS T3 column (100 × 2.1 mm, 1.8 μm). The mobile phases consisted of water containing 0.1% formic acid (A) and acetonitrile containing 0.1% formic acid (B). The flow rate was 0.4 mL/min, and the injection volume was 2 μL.

The gradient program was as follows: 0–0.5 min, 5% B; 0.5–5.5 min, 5–50% B; 5.5–9.0 min, 50–95% B; 9.0–10.5 min, 95% B; and 10.5–12.0 min, 95–5% B.

Mass-spectrometric data were acquired in negative-ion electrospray ionization mode using data-independent MSE acquisition under MassLynx v4.2 control. Spectra were acquired over an *m/z* range of 50–1200 with a scan time of 0.2 s per spectrum. The mass spectrometer was operated at a resolving power of approximately 30,000 full width at half maximum under the acquisition conditions used in this study. The low-energy function was acquired at 2 eV, whereas the elevated-energy function used a collision-energy ramp of 10–40 eV.

The capillary voltage was −2.0 kV, the cone voltage was 30 V, the source temperature was 100 °C, and the desolvation temperature was 500 °C. The cone-gas and desolvation-gas flow rates were 50 and 800 L/h, respectively. Pooled QC samples were injected periodically within each analytical batch to monitor signal stability and analytical reproducibility.

#### 2.4.3 Data processing and metabolite annotation

Raw LC–MS data acquired using MassLynx v4.2 were processed separately for each analytical batch using Progenesis QI v4.0 (Waters Corporation, Newcastle upon Tyne, UK) for peak detection, retention-time alignment, peak integration, and feature-table generation. Peak areas were normalised to the total peak area within each analytical batch before subsequent statistical analysis.

Metabolite features were putatively annotated by matching precursor-ion accurate masses, isotope distributions, theoretical fragment ions, and elevated-energy MSE spectra against the online METLIN database, other public databases, and an in-house spectral library. The mass deviation allowed for database matching and theoretical-fragment assignment was ≤100 ppm. Because authentic reference standards were not systematically used for confirmation, the assignments were reported as putatively annotated metabolite features, primarily corresponding to Metabolomics Standards Initiative Level 2 or Level 3, rather than definitively identified Level 1 metabolites.

Principal component analysis and Spearman correlation analysis were used to evaluate overall sample distribution, bottle-level replicate consistency, and pooled-QC reproducibility within each analytical batch. Pairwise orthogonal partial least-squares discriminant analysis models were constructed using the R package ropls v1.6.2, and model reliability was assessed using 200 permutation tests. Variable-importance-in-projection values were obtained from the OPLS-DA models. Differential metabolite features were selected using VIP ≥ 1 and *P* < 0.05 based on univariate *t*-tests. Fold-change values were used to indicate only the direction and magnitude of variation and were not applied as an additional selection threshold.

Because the CK samples were analysed in a separate analytical batch, the LC–MS feature tables from CK and FW/PW were not directly merged for cross-batch differential modeling. The differential non-volatile metabolomic analyses presented in this study were therefore restricted to stage-matched comparisons between PW and FW within the same analytical batch: EP versus EF, MP versus MF, and LP versus LF. KEGG pathway-enrichment analysis of the differential features was performed using a hypergeometric test.

### 2.5 Volatile profiling of *Phyllanthus emblica* L. fruit wines by HS-SPME–GC–MS

Volatile compounds were analysed by headspace solid-phase microextraction coupled with gas chromatography–mass spectrometry (HS-SPME–GC–MS). Frozen wine samples were thawed and thoroughly homogenized, and 700 ± 5 mg of each sample was transferred into a 20 mL headspace vial. Subsequently, 10 μL of a 2-octanol solution (10 mg/ L in deionized water) was added as an internal reference. The 2-octanol signal was used to monitor extraction and instrumental consistency but was not used for absolute quantification.

The samples were pre-equilibrated at 60 °C for 15 min using a PAL Rail automated sampling system. Volatile compounds were then extracted from the headspace at 60 °C for 30 min using a 120 μm divinylbenzene/carbon wide range/polydimethylsiloxane (DVB/CWR/PDMS) SPME Arrow and thermally desorbed in the GC inlet at 250 °C for 4 min.

GC–MS analysis was performed using an Agilent 7890B gas chromatograph coupled to an Agilent 5977B mass-selective detector (Agilent Technologies, Santa Clara, CA, USA). Chromatographic separation was achieved using a DB-Wax capillary column (30 m × 0.25 mm i.d., 0.25 μm film thickness; Agilent J&W Scientific, Folsom, CA, USA). The SPME Arrow was introduced into the inlet in splitless mode. Helium was used as the carrier gas at a constant column flow rate of 1.0 mL/ min, and the inlet septum-purge flow was 3.0 mL/min. The oven temperature was maintained at 40 °C for 4 min, increased to 245 °C at 5 °C min^−1^, and held at 245 °C for 5 min. The inlet, transfer-line, ion-source, and quadrupole temperatures were 250, 250, 230, and 150 °C, respectively. Mass spectra were acquired in full-scan electron-ionization mode at 70 eV over an *m/z* range of 20–400, with a solvent delay of 0 min. Raw data were processed using ChromaTOF v4.3× (LECO Corporation, St. Joseph, MI, USA) for peak extraction, baseline correction, spectral deconvolution, retention-time alignment, peak integration, and mass-spectral-library searching. Volatile features were tentatively annotated by matching their electron-ionization mass spectra against the NIST20 mass-spectral library. Only features with library-matching scores of ≥80% that passed manual plausibility screening were retained for subsequent analysis. Assignments that were not confirmed using authentic reference standards and experimentally determined retention indices were considered tentative.

Obvious background contaminants, analytical artifacts, siloxanes, column-bleed products, implausible library assignments, and the 2-octanol internal-reference peak were excluded before normalization. The peak areas of the remaining sample-derived volatile features were converted to relative peak-area percentages using total-area normalization:

$$R_{i}\left(\%\right)=\frac{A_{i}}{\sum_{j=1}^{n}A_{j}}\times 100$$

where *Ri* is the relative peak-area percentage of volatile feature i, *Ai* is the peak area of feature i, and $\sum_{j=1}^{n}A_{j}$ is the sum of the peak areas of all *n* retained volatile features after exclusion of the internal-reference peak, background contaminants, analytical artifacts, and chemically implausible assignments (Liu, Qiao, et al., 2024). Because compound-specific calibration curves and response factors were not established, the resulting relative peak-area percentages represent compositional proportions rather than absolute concentrations or absolute compound amounts.

### 2.6 Statistical analysis

Data are presented as mean ± standard deviation of three bottle-level fermentation replicates randomly selected and destructively sampled at each treatment–stage combination (*n* = 3). For comparisons involving more than two groups, univariate data were analysed by one-way analysis of variance followed by Duncan's multiple-range test using IBM SPSS Statistics 26.0 (IBM Corp., Armonk, NY, USA). Differences were considered statistically significant at *p <* 0.05.

Microbial community patterns were visualised by principal component analysis (PCA) based on operational taxonomic unit (OTU) relative-abundance profiles. β-Diversity was evaluated using binary Jaccard and Bray–Curtis distance matrices. Differences among treatment–stage combinations were assessed by permutational multivariate analysis of variance (PERMANOVA) and analysis of similarities (ANOSIM) implemented using the vegan package in R; the PERMANOVA and ANOSIM results reported in this study were based on binary Jaccard distances. Alpha- and β-diversity analyses were performed using QIIME 2 and the BMKCloud platform, as described in Section 2.3.

Non-volatile metabolomic and volatile-profile datasets were subjected to PCA and pairwise orthogonal partial least-squares discriminant analysis (OPLS-DA) after unit-variance scaling using the ropls package in R. OPLS-DA model reliability was evaluated by cross-validation and 200 permutation tests. For each pairwise comparison, univariate *p* values were calculated using two-sided Student's *t*-tests. Features with variable-importance-in-projection (VIP) values ≥1 and nominal *p <* 0.05 were considered differential non-volatile metabolite features or differential volatile features. Fold-change values were used to describe the direction and magnitude of variation but were not applied as an additional primary screening threshold.

For the more stringently screened and chemically curated FW-versus-PW volatile-feature subset presented in Table S2, the additional criteria of Benjamini–Hochberg FDR-adjusted *p <* 0.05 and ∣log_2_FC∣ ≥ 1 were applied, followed by chemical-plausibility screening and the exclusion of probable background contaminants and analytical artifacts. Fold change was calculated as FW/PW.

LC–MS-based non-volatile metabolomic data were preprocessed and statistically analysed within their respective analytical batches. Because the CK samples were analysed in a separate LC–MS batch, differential non-volatile metabolomic comparisons were restricted to FW and PW samples analysed under identical analytical conditions. Figures were prepared using Origin 2021 (OriginLab Corporation, Northampton, MA, USA).

## 3 Results and discussion

### 3.1 Stage-associated changes in microbial communities during *Phyllanthus emblica* L. fruit wine fermentation

The bacterial and fungal feature-accumulation curves approached asymptotes as the number of samples increased, suggesting that the current sample set captured most of the detectable community features in this dataset (Fig. 1a and e). In these plots, the red boxes represent the total number of features detected in randomly selected sample subsets, whereas the green boxes represent the number of features shared among the selected samples. Flower plots showed that 279 bacterial OTUs and six fungal OTUs were shared among all nine treatment–stage combinations, together with numerous combination-specific OTUs (Fig. 1b and f). These patterns indicated substantial stage- and treatment-associated variation in bacterial and fungal community composition.

**Fig. 1:**
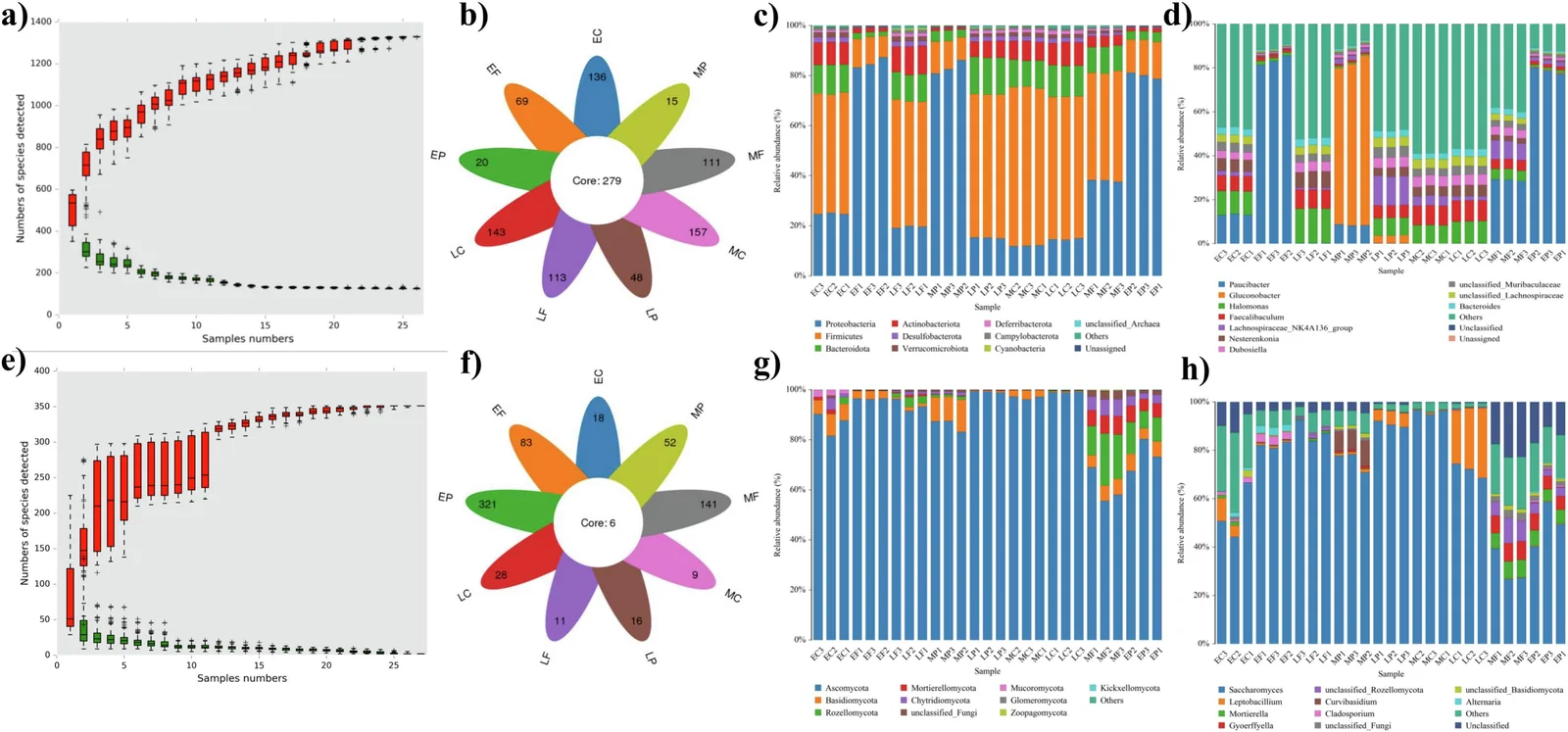
Bacterial and fungal community profiles during *Phyllanthus emblica* L. fruit wine fermentation. OTU-accumulation curves for bacterial (a) and fungal (e) datasets, with red and green boxplots representing cumulative and shared OTU richness, respectively, across randomly selected sample subsets. Flower plots illustrate OTUs shared among all nine treatment–stage groups and OTUs detected exclusively in individual groups for bacteria (b) and fungi (f). Relative abundances are shown at the bacterial phylum (c) and genus (d) levels and the fungal phylum (g) and genus (h) levels. Each bar represents one bottle-level fermentation replicate. E, M, and L denote early, middle, and late fermentation stages; F, P, and C denote FW, PW, and CK, respectively. FW, juice wine fermented at 20 °C; PW, pomace-containing juice wine fermented at 20 °C; CK, juice wine fermented at 26 °C. (For interpretation of the references to colour in this figure legend, the reader is referred to the web version of this article.)

At the phylum level, Proteobacteria and Firmicutes dominated the bacterial communities and together accounted for more than 69% of the relative abundance in all samples (Fig. 1c). In FW, the bacterial profile shifted from Proteobacteria predominance at the early stage toward greater contributions from Firmicutes and Bacteroidota at the middle and late stages. In PW, Proteobacteria remained predominant at the early and middle stages but decreased markedly at the late stage, accompanied by increases in Firmicutes and Bacteroidota. By contrast, Firmicutes remained the predominant bacterial phylum throughout CK fermentation. Ascomycota was the dominant fungal phylum in all treatments, with relative abundances exceeding 57% (Fig. 1g). Basidiomycota, Rozellomycota, and Mortierellomycota showed greater stage-associated variation, particularly in the middle and late stages of FW and PW. These results describe compositional changes and do not provide direct evidence of physiological adaptation of individual phyla to fermentation temperature.

At the genus level, *Paucibacter* predominated in EF and EP, accounting for 79.14% and 77.18% of the bacterial sequences, respectively (Fig. 1d). In MP, *Gluconobacter* reached a relative abundance of 81.84%. This marked enrichment was associated with the middle stage of the pomace-containing fermentation; however, DNA-based sequencing alone cannot establish the viability, metabolic activity, or functional contribution of *Gluconobacter*. Grape-associated *Gluconobacter* has also been detected during wine fermentation, although its functional importance depends on strain activity and fermentation conditions (Wu et al., 2025).

*Saccharomyces* was the predominant fungal genus in most samples (Fig. 1h). In FW, its relative abundance was high in EF and LF but lower in MF. In PW, the relative contribution of *Saccharomyces* increased from EP to MP and was highest in LP. In CK, its relative abundance varied among stages, with particularly high values in MC. The widespread predominance of *Saccharomyces* is consistent with vinification studies showing that inoculated *S. cerevisiae* frequently becomes a major component of the fungal community during alcoholic fermentation (Bekris et al., 2024). Stage-associated fungal-community changes have also been reported in kiwifruit, blueberry, passion-fruit, and pomelo wines using amplicon sequencing (Hu et al., 2023; Wu et al., 2026; Ye et al., 2023; Zhang et al., 2022).

The yeast-count dynamics observed in our previous fermentation-kinetics study showed that the monitored yeast population increased during early fermentation and subsequently stabilized or decreased as fermentation progressed (Liu et al., 2026). Nevertheless, these measurements do not establish the viability of every bacterial or fungal taxon detected by amplicon sequencing. Accordingly, DNA-derived relative abundances were interpreted as compositional community profiles rather than direct measures of microbial viability, proliferation, or metabolic activity.

PCA revealed distinct positioning of several treatment–stage combinations, although overlap remained among some groups (Figs. 2a and 3a). For the bacterial communities, PC1 and PC2 explained 65.09% and 34.14% of the total variation, respectively, giving a cumulative contribution of 99.23%. For the fungal communities, PC1 and PC2 explained 84.42% and 11.47%, respectively, with a cumulative contribution of 95.89%. Replicates from most treatment–stage combinations clustered closely in the hierarchical analyses, indicating relatively consistent community profiles within groups (Figs. 2b and 3b).

**Fig. 2:**
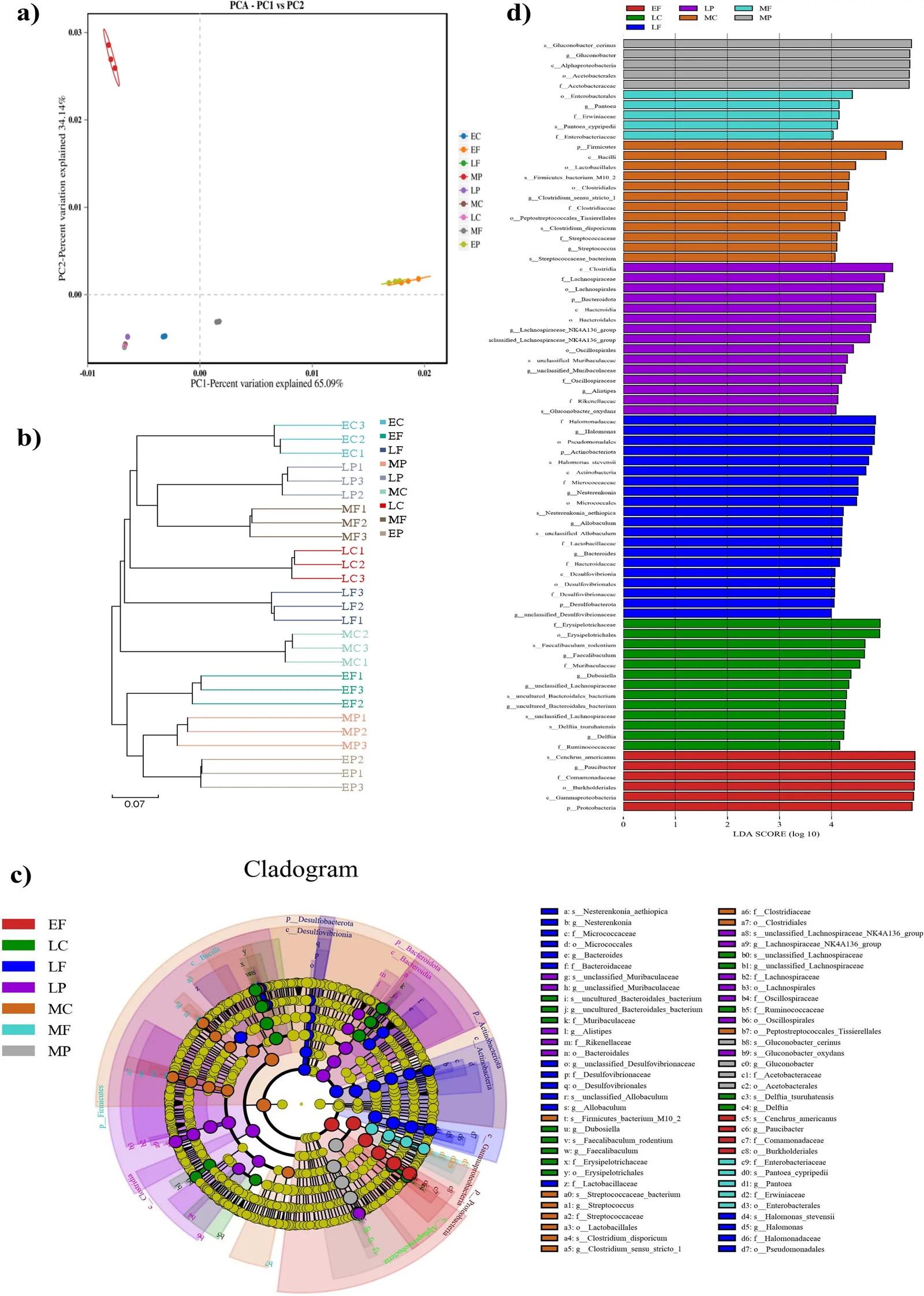
Bacterial community structure and discriminatory taxa during *Phyllanthus emblica* L. fruit wine fermentation. (a) Principal coordinate analysis (PCA) based on bacterial OTU relative-abundance profiles; (b) hierarchical clustering of bacterial community profiles; (c) linear discriminant analysis effect size (LEfSe) cladogram showing discriminatory taxonomic lineages among treatment–stage combinations; and (d) linear discriminant analysis (LDA) scores of discriminatory taxa identified using an LDA score > 4.0. Each point in panel a and each terminal label in panel b represents one bottle-level fermentation replicate. E, M, and L indicate the early, middle, and late fermentation stages, respectively. F, P, and C denote FW, PW, and CK, respectively. FW, juice wine fermented at 20 °C; PW, pomace-containing juice wine fermented at 20 °C; CK, juice wine fermented at 26 °C. Prefixes p_, c_, o_, f_, g_, and s_ denote phylum, class, order, family, genus, and species, respectively.

**Fig. 3:**
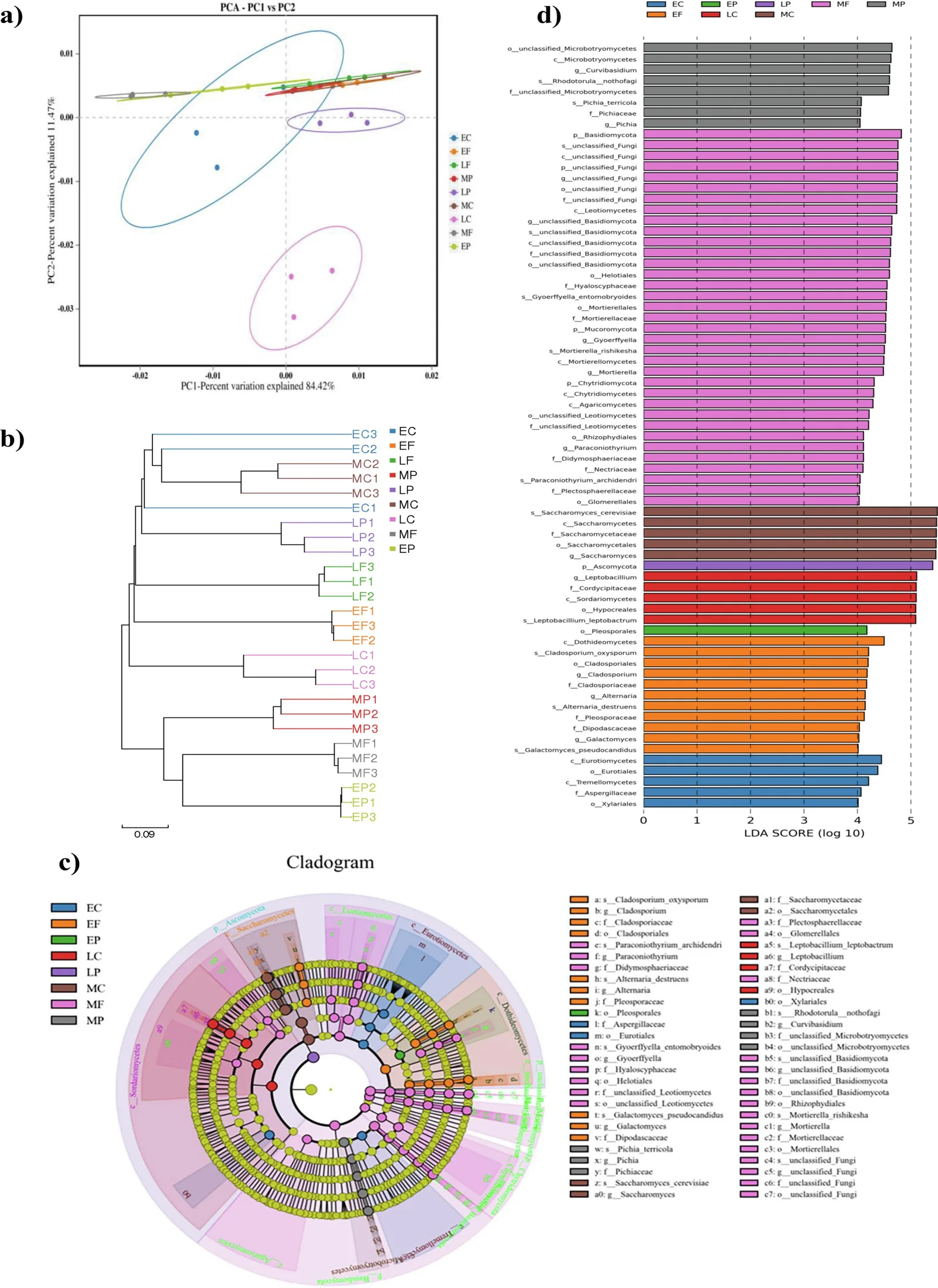
Fungal community structure and discriminatory taxa during *Phyllanthus emblica* L. fruit wine fermentation. (a) Principal coordinate analysis (PCA) of fungal OTU relative-abundance profiles; (b) hierarchical clustering of fungal community profiles; (c) linear discriminant analysis effect size (LEfSe) cladogram showing discriminatory taxonomic lineages among the treatment–stage combinations; and (d) linear discriminant analysis (LDA) scores of discriminatory fungal taxa identified with an LDA score > 4.0. Each point in panel a and each terminal label in panel b represents one bottle-level fermentation replicate. Sample codes combine fermentation stage (E, early; M, middle; L, late) and treatment (F, FW; P, PW; C, CK). FW, juice wine fermented at 20 °C; PW, pomace-containing juice wine fermented at 20 °C; CK, juice wine fermented at 26 °C. Prefixes p_, c_, o_, f_, g_, and s_ denote phylum, class, order, family, genus, and species, respectively.

Global differences among the nine treatment–stage combinations were further supported by binary-Jaccard-based PERMANOVA and ANOSIM. For the bacterial community, PERMANOVA yielded a pseudo-*F* value of 15.66, an *R*^*2*^ of 0.874, and a *P* value of 0.001, whereas ANOSIM yielded an *R* value of 1.000 and a *P* value of 0.001. For the fungal community, PERMANOVA yielded a pseudo-*F* value of 16.49, an *R*^*2*^ of 0.880, and a *P* value of 0.001, whereas ANOSIM yielded an *R* value of 0.967 and a *P* value of 0.001. PCA and hierarchical clustering were therefore used for visualisation, whereas the global statistical differences among groups were evaluated using the distance-based tests.

LEfSe analysis using an LDA threshold of 4.0 identified discriminatory bacterial taxa associated with individual treatment–stage combinations (Fig. 2c and d). *Paucibacter* and members of Burkholderiales and Comamonadaceae were associated with EF, whereas *Gluconobacter*, Acetobacteraceae, and Acetobacterales characterised MP. *Pantoea* and Enterobacterales were associated with MF, while Lactobacillales, *Streptococcus*, and *Clostridium* sensu stricto were discriminatory taxa in MC. Lachnospiraceae, *Bacteroides*, and Oscillospirales were mainly associated with LP. These taxa represent stage-associated discriminatory signatures rather than stable treatment-wide biomarkers.

The fungal LEfSe analysis similarly revealed stage-associated taxonomic signatures (Fig. 3c and d). *Cladosporium*, *Alternaria*, and *Galactomyces* were associated mainly with EF; *Pichia* and *Rhodotorula* characterised MP; and *Mortierella* and related Mortierellales taxa were associated with MF. *Saccharomyces* and Saccharomycetaceae were discriminatory taxa in MC, whereas *Leptobacillium* and members of Hypocreales were associated with LC. These taxa are described as discriminatory taxonomic signatures rather than key functional microorganisms because LEfSe identifies relative-abundance differences but does not verify metabolic function. Recent wine studies have also combined microbial sequencing with metabolite analysis to investigate associations between microbial succession and flavour profiles, although such relationships remain correlative without strain isolation and controlled-fermentation validation (Ding et al., 2024).

Overall, Fig. 1, Fig. 2, Fig. 3 show pronounced stage- and treatment-associated restructuring of bacterial and fungal community profiles during *P. emblica* fruit wine fermentation. The enrichment of *Gluconobacter* in MP and the stage-associated variation in *Saccharomyces* were among the most evident compositional features. However, because the experimental design did not include a pomace-containing treatment fermented at 26 °C, the independent effects of fermentation temperature and pomace addition cannot be separated. Moreover, DNA-based amplicon sequencing detects DNA from both viable and non-viable cells and is affected by compositional and amplification biases. The results therefore should not be interpreted as direct evidence of microbial proliferation, metabolic activity, or causal contributions to flavour formation (Zhao & Gänzle, 2025).

### 3.2 Non-volatile metabolite profiles during fermentation of *Phyllanthus emblica* L. fruit wine

#### 3.2.1 Stage-associated patterns of major non-volatile metabolite classes

Untargeted UHPLC–QTOF–MS analysis generated 3964 aligned LC–MS ion features across the FW and PW samples, of which 2936 received tentative metabolite annotations. Following manual review of the database-derived classifications, the annotated features comprised organic acids and related compounds (1425), sugars and their derivatives (622), fatty acids and related lipids (426), amino acids and their derivatives (403), a database-derived alcohol-related category (38), and phenolic compounds (22). Each heatmap cell in Fig. 4 represents the row-wise standardised mean signal intensity of three bottle-level fermentation replicates for the corresponding treatment–stage group. The heatmaps therefore show relative differences among groups rather than absolute metabolite concentrations. Comparable fruit-wine studies have also reported fermentation-associated changes in carbohydrates, organic acids, amino acids, lipids, and phenolic compounds (Jiang et al., 2024; Li et al., 2025; Zhao et al., 2024).

**Fig. 4:**
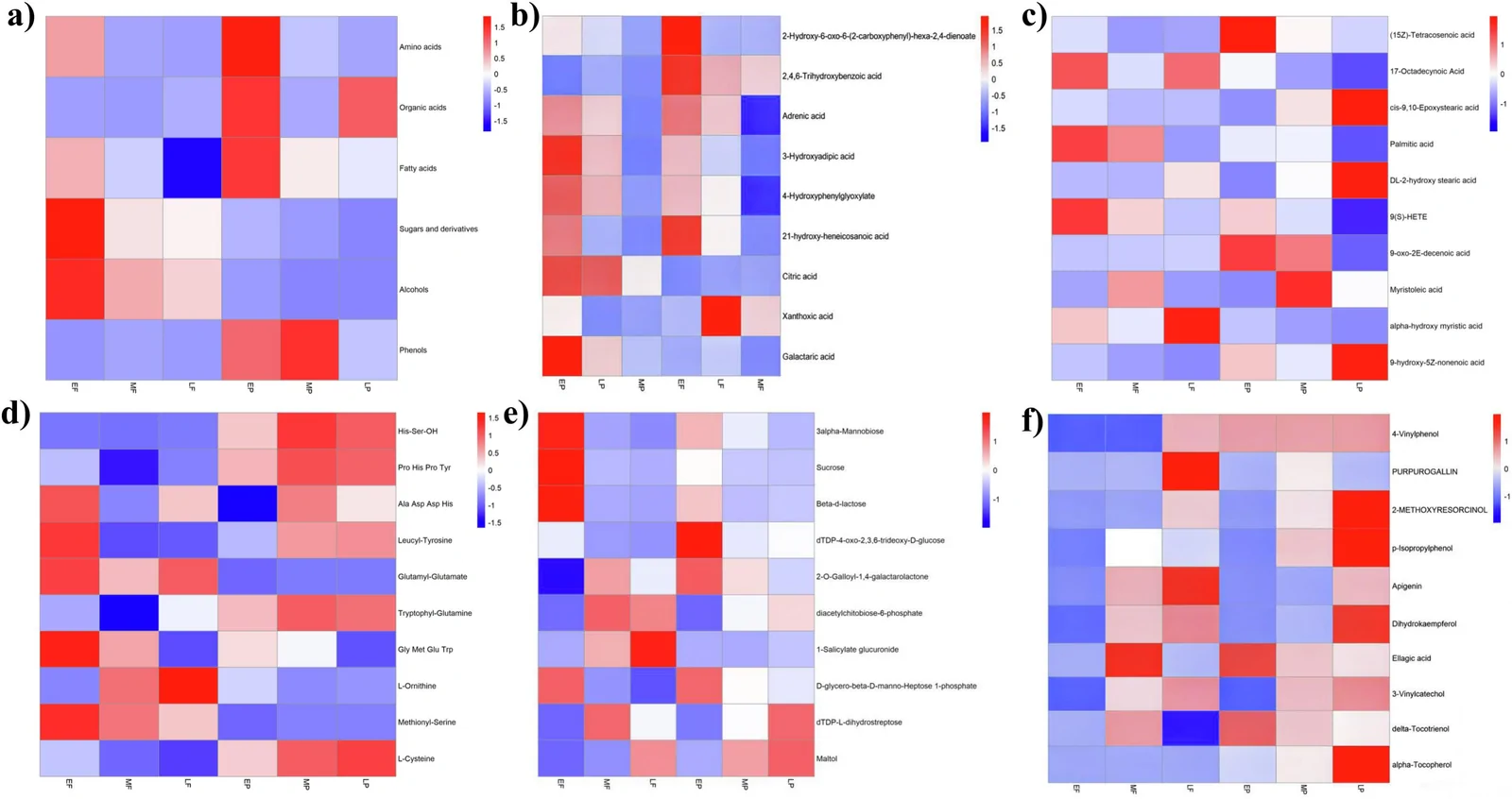
Stage-associated patterns of non-volatile metabolite features in FW and PW during fermentation at 20 °C. (a) Major chemical classes of putatively annotated non-volatile metabolite features; and heatmaps of selected features putatively annotated as organic acids and related compounds (b), fatty acids and related lipids (c), amino acids and derivatives (d), sugars and derivatives (e), and phenolic compounds (f). Red and blue indicate relatively higher and lower standardised signals after row-wise z-score normalization, respectively. EF, MF, and LF denote the early, middle, and late stages of FW fermentation, respectively; EP, MP, and LP denote the corresponding stages of PW fermentation. (For interpretation of the references to colour in this figure legend, the reader is referred to the web version of this article.)

The major database-derived chemical classes and their stage- and treatment-associated patterns are summarised in Fig. 4a. Sugar-related signals were generally higher at the early stages and lower at the middle and late stages. Organic-acid-, amino-acid-, lipid-, and phenolic-related features also differed between FW and PW, indicating pomace-associated variation under the same fermentation temperature of 20 °C. The database-derived alcohol-related category was retained only in the overall classification summary and was not interpreted further because it contained chemically heterogeneous and tentatively annotated features.

Organic-acid-related features, including tentative assignments to citric acid, galactaric acid, hydroxy-acid derivatives, and other carboxylic-acid derivatives, varied among the treatment–stage groups (Fig. 4b). These differences may reflect variation in precursor availability, extraction from the pomace matrix, and fermentation-associated transformation. However, the identities and formation pathways of individual features require confirmation using authentic standards and targeted analysis.

Fatty-acid- and amino-acid-related features also showed stage-associated variation (Fig. 4c and d). Representative tentative assignments included tetracosenoic acid, palmitic acid, L-ornithine, L-citrulline, methionyl-serine, and histidyl-serine. Amino-acid metabolism has similarly been identified as an important source of variation in fruit-wine metabolomic studies (Jiang et al., 2024; Li et al., 2025). However, standardised LC–MS signals do not directly establish nutritional availability or sensory contribution.

Features tentatively assigned to mannobiose, sucrose, lactose-related structures, and other carbohydrate derivatives generally showed lower signals at the middle and late stages than at the corresponding early stages (Fig. 4e), consistent with carbohydrate utilization and transformation.

Phenolic-related features, including tentative assignments to apigenin, dihydrokaempferol, vinylcatechol, ellagic acid, and tocopherol-related compounds, also varied between treatments and fermentation stages (Fig. 4f). Because these assignments were not systematically confirmed using authentic standards, their biological, nutritional, antioxidant, and sensory functions were not inferred directly from the standardised signal patterns.

#### 3.2.2 Pomace-associated differential features and pathway enrichment

The tentatively annotated non-volatile metabolite features were mapped to 47 KEGG pathways, of which the 20 pathways containing the largest numbers of mapped features are shown in Fig. 5a. Major categories included amino-acid, carbohydrate, lipid, cofactor and vitamin, and secondary-metabolite metabolism. UHPLC–QTOF–MS combined with multivariate analysis and KEGG mapping has also been used to characterize metabolic variation in wines and other fermented fruit beverages (Yin et al., 2024; Zhao et al., 2024).

**Fig. 5:**
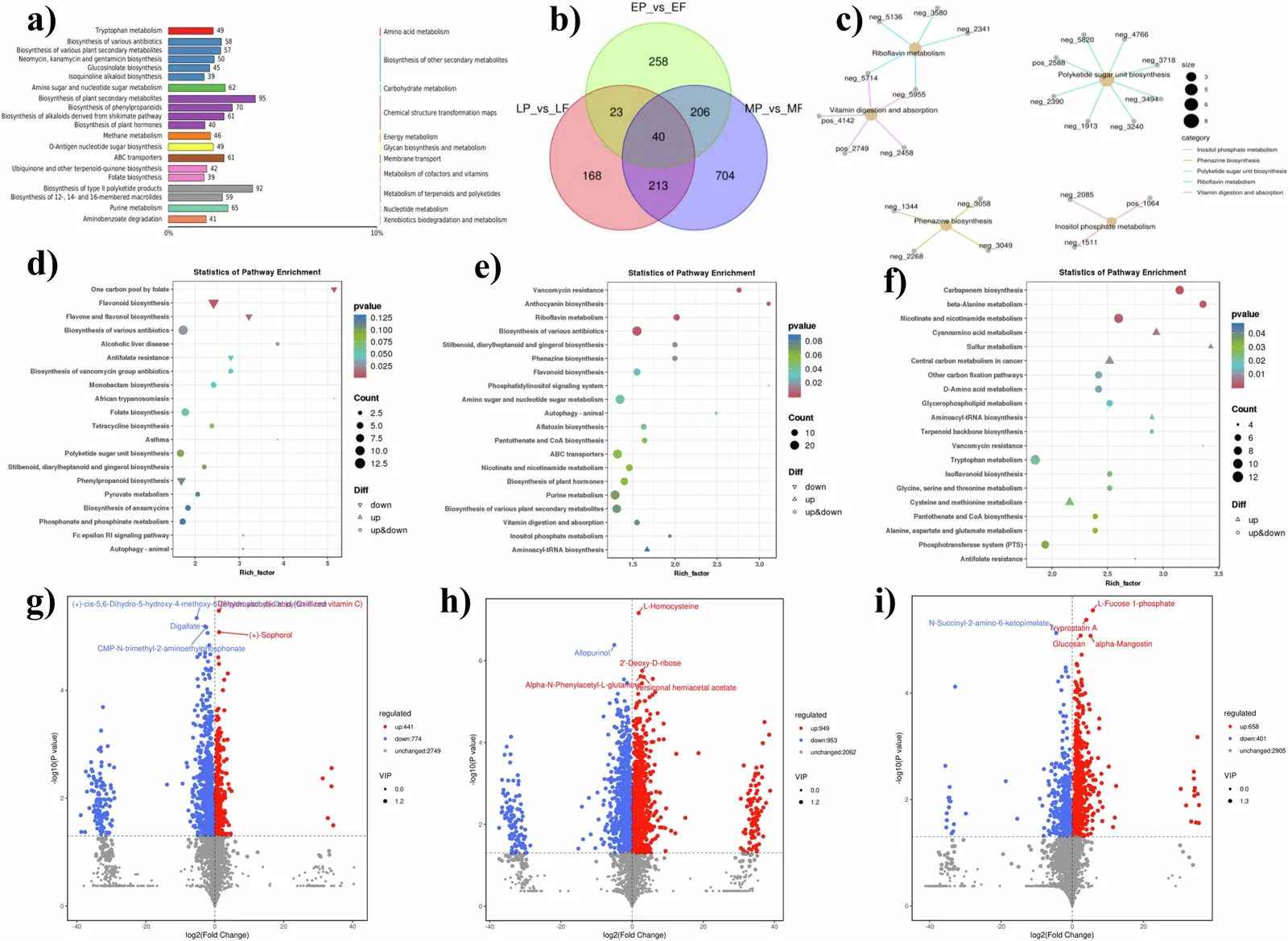
Differential non-volatile metabolite features associated with pomace addition during fermentation at 20 °C. (a) KEGG classification of KEGG-mapped putatively annotated features; (b) Venn diagram showing shared and comparison-specific putatively annotated differential features; (c) pathway–feature network based on the KEGG-mapped subset of the 40 differential features shared among EP versus EF, MP versus MF, and LP versus LF comparisons; (d–f) KEGG enrichment plots based on KEGG-mapped differential features; and (g–i) volcano plots for EP versus EF, MP versus MF, and LP versus LF comparisons, respectively. The volcano plots include all 3964 aligned LC–MS ion features, whereas the Venn diagram includes only the putatively annotated differential-feature subset. In panels d–f, symbol size, colour, and shape indicate mapped-feature number, enrichment *p* value, and regulation direction, respectively. In panels g–i, red, blue, and grey indicate features with higher, lower, and non-differential signals in PW relative to FW; point size represents VIP values. Differential features were selected using VIP ≥1 and *p* < 0.05. (For interpretation of the references to colour in this figure legend, the reader is referred to the web version of this article.)

Differential-feature analysis compared PW with FW at matched fermentation stages: EP versus EF, MP versus MF, and LP versus LF. Thus, Fig. 5 characterizes pomace-associated differences under the same fermentation temperature of 20 °C and does not include CK. Forty annotated differential features were shared among the three comparisons, whereas MP versus MF contained the largest number of comparison-specific features (Fig. 5b). This finding indicates that the middle-stage comparison contained the greatest number of uniquely selected features but does not establish a greater overall degree of metabolic differentiation.

KEGG enrichment patterns varied among fermentation stages (Fig. 5d–f). The early-stage comparison included pathways related to the one‑carbon pool by folate and flavonoid biosynthesis, whereas the middle- and late-stage comparisons included riboflavin metabolism, β-alanine metabolism, carbohydrate- and amino-acid-related pathways, and secondary-metabolite biosynthesis. Riboflavin-related features may be associated with flavin-dependent redox processes, whereas inositol-phosphate-related features may reflect variation in phosphorylated carbohydrate metabolism. However, pathway enrichment indicates coordinated differences among mapped features rather than increased pathway activity or metabolic flux.

Volcano plots showed differential-feature patterns between PW and FW at all three stages (Fig. 5g–i). The labelled features mainly included tentative assignments related to organic acids, carbohydrates, amino-acid derivatives, and phenolic compounds. The 40 shared differential features were mapped to five interconnected KEGG pathways: phenazine biosynthesis, riboflavin metabolism, inositol phosphate metabolism, polyketide sugar-unit biosynthesis, and vitamin digestion and absorption (Fig. 5c). These associations suggest coordinated variation in redox-cofactor-, carbohydrate-phosphate-, and secondary-metabolite-related processes. Antibiotic-related pathway labels, including vancomycin resistance and carbapenem biosynthesis, were not interpreted as evidence of antibiotic resistance or production because individual metabolites may be assigned to multiple KEGG pathways.

Overall, pomace addition was associated with stage-dependent differences in non-volatile metabolite profiles during fermentation at 20 °C. Because the assignments were based mainly on accurate mass, theoretical fragmentation, and database matching, the feature identities and pathway associations should be regarded as tentative. Annotation uncertainty remains an important limitation of untargeted metabolomics, particularly for isomers, redundant ion features, and compounds lacking reference spectra (Zhang, Zeng, et al., 2025).

### 3.3 Volatile profiles of final-stage *Phyllanthus emblica* L. fruit wines

#### 3.3.1 Composition of volatile profiles

HS-SPME–GC–MS profiling yielded 256 tentatively annotated volatile features in the combined final-stage FW and PW dataset. Of these, 249 features were shared between the two treatments, whereas six and one were detected exclusively in FW and PW, respectively (Fig. 6b). Thus, FW and PW exhibited broadly similar qualitative volatile-feature profiles under the same fermentation temperature of 20 °C, although the proportional composition of these profiles differed.

**Fig. 6:**
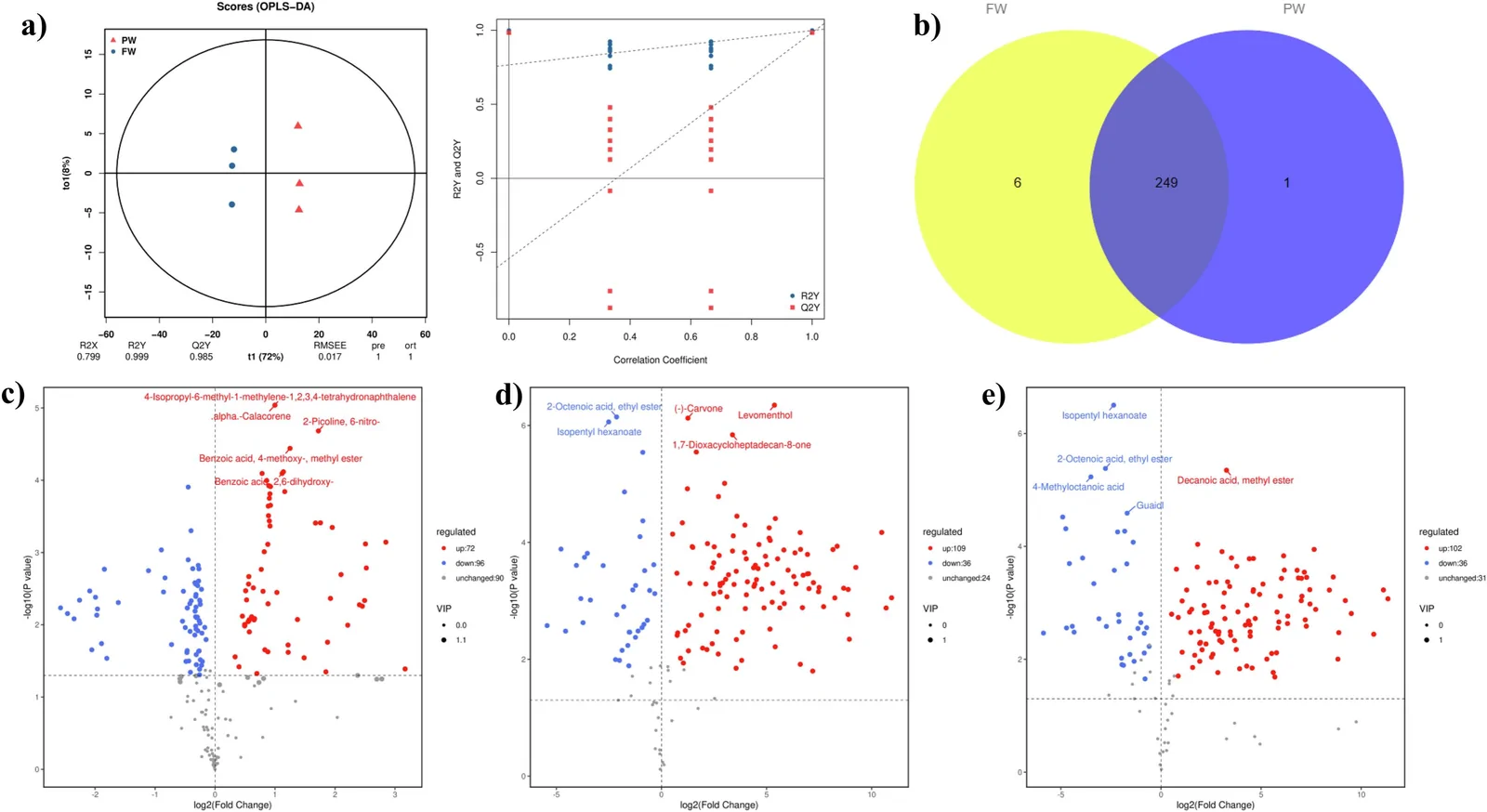
Multivariate and differential analyses of volatile-feature profiles in final-stage *Phyllanthus emblica* L. fruit wines. (a) OPLS-DA score plot distinguishing FW and PW and the corresponding permutation validation plot; (b) Venn diagram showing shared and treatment-specific detected volatile-feature records in final-stage FW and PW; and volcano plots comparing FW versus PW (c, log₂[FW/PW]), FW versus CK (d, log₂[FW/CK]), and PW versus CK (e, log₂[PW/CK]). Panel b summarizes the 256 detected volatile-feature records in the combined final-stage FW and PW dataset, including 249 shared records, six records detected exclusively in FW, and one record detected exclusively in PW. Panels c–e present differential analyses based on the aligned volatile-feature records subjected to statistical testing. Red and blue indicate features with higher and lower normalised responses, respectively, in the first group named in each comparison, whereas grey indicates non-differential features. Point size represents VIP values. Differential features were selected using VIP ≥ 1 and nominal *p <* 0.05. (For interpretation of the references to colour in this figure legend, the reader is referred to the web version of this article.)

After exclusion of the 2-octanol internal-reference peak, background contaminants, analytical artifacts, and chemically implausible library assignments, 242 retained volatile features were included in the compositional analysis (Table 1). The complete feature-level data and tentative GC–MS library annotations for these retained volatile features are provided in Table S1. Alcohols constituted the largest chemical class in both treatments, accounting for 39.15 ± 0.88% and 30.28 ± 0.95% of the FW and PW profiles, respectively. Esters represented 12.27 ± 0.29% in FW and 10.50 ± 0.43% in PW, whereas alkanes, alkenes, and terpenoids accounted for 15.12 ± 0.37% and 12.29 ± 0.56%, respectively. In contrast, ethers represented a substantially larger proportion in PW than in FW (22.98 ± 0.83% versus 6.90 ± 0.20%). Aldehydes accounted for similar proportions in FW and PW, whereas ketones, acids, phenols, and other compounds constituted smaller fractions of both profiles.

**Table 1:** Relative peak-area percentages of major volatile-feature classes and representative tentative library assignments in low-temperature-fermented *Phyllanthus emblica* L. juice wine and pomace-containing juice wine.

Class	Class relative peak-area percentage in FW (%)	Class relative peak-area percentage in PW (%)	Representative tentative library assignments	Feature relative peak-area percentage in FW (%)	Feature relative peak-area percentage in PW (%)
Esters	12.27 ± 0.29	10.50 ± 0.43	Ethanethioic acid, S-(2-furanylmethyl) ester	2.67 ± 0.14	2.56 ± 0.22
			Ethyl 3-(methylthio)-(E)-2-propenoate	1.09 ± 0.33	0.75 ± 0.07
			(*S*)-3,3,6-Trimethylhepta-1,5-dien-4-yl acetate	1.01 ± 0.09	0.99 ± 0.10
Alcohols	39.15 ± 0.88	30.28 ± 0.95	2-Pentanol	15.50 ± 0.90	6.76 ± 4.36
			Ethanol, 2-(2-butoxyethoxy)-	5.95 ± 0.51	4.93 ± 3.85
			(6Z)-Nonen-1-ol	4.83 ± 0.28	4.71 ± 0.40
Ketones	7.97 ± 0.35	7.10 ± 0.32	1-Nonen-3-one	0.57 ± 0.03	0.62 ± 0.06
			3-Hydroxy-3-methyl-2-butanone	1.17 ± 0.01	0.71 ± 0.87
Acids	3.07 ± 0.09	2.47 ± 0.05	Octanoic acid	0.58 ± 0.06	0.49 ± 0.05
			2-Methylheptanoic acid	0.49 ± 0.02	0.53 ± 0.05
Phenols	0.71 ± 0.01	0.55 ± 0.06	Phenol, 3,4-dimethoxy-	0.12 ± 0.00	0.07 ± 0.01
			Phenol, 2,6-dimethoxy-	0.12 ± 0.00	0.07 ± 0.01
Ethers	6.90 ± 0.20	22.98 ± 0.83	Benzene, (2-methoxyethyl)-	6.67 ± 0.20	22.76 ± 4.22
Aldehydes	7.13 ± 0.12	7.09 ± 0.58	2-Undecenal	1.43 ± 0.01	0.88 ± 0.07
			Pentanal	1.06 ± 0.01	1.71 ± 0.11
Alkanes, alkenes, and terpenoids	15.12 ± 0.37	12.29 ± 0.56	Naphthalene, 1,2-dihydro-1,1,6-trimethyl-	2.26 ± 0.06	0.90 ± 0.07
			Cyclohexene, 1-methyl-4-(1-methylethylidene)-	0.32 ± 0.01	1.13 ± 0.18
Other compounds	7.68 ± 0.19	6.72 ± 0.60	Thiophene, 2-pentyl-	0.54 ± 0.02	0.53 ± 0.04

Values are expressed as mean ± standard deviation (*n* = 3 independent bottle-level fermentation replicates). FW and PW were sampled on days 15 and 13, respectively. Before total-area normalization, the 2-octanol internal-reference peak, background contaminants, analytical artifacts, and chemically implausible assignments were excluded. Chemical-class percentages were calculated by summing the relative peak-area percentages of all 242 retained volatile features assigned to each class; therefore, the representative assignments listed do not necessarily sum to the corresponding class percentage. Only features with NIST20 library-match scores ≥80% that passed plausibility screening were retained. Assignments not confirmed using authentic standards and experimentally determined retention indices are tentative. Values represent proportional volatile-profile composition rather than absolute concentrations. Complete feature-level data are provided in Table S1, whereas Table S2 presents statistical metrics for a more stringently screened and chemically curated subset of differential FW-versus-PW features. Values are rounded to two decimal places, and percentages may not sum exactly to 100%.

The values reported in Table 1 are relative peak-area percentages obtained by total-area normalization and therefore describe compositional proportions within the detected volatile profiles rather than absolute concentrations or compound amounts. Moreover, the listed feature assignments were based on NIST20 electron-ionization mass-spectral-library matching and were not confirmed using authentic standards or experimentally determined retention indices; they should therefore be regarded as tentative. Because aroma perception depends on compound concentration, odour threshold, and interactions within the volatile matrix, these proportional data alone do not establish aroma intensity, complexity, or quality (Zang et al., 2025; Zhao et al., 2024).

#### 3.3.2 Multivariate and differential analysis of volatile features

OPLS-DA differentiated the final-stage volatile profiles of FW and PW (Fig. 6a), indicating that pomace addition was associated with variation in volatile composition under the same fermentation temperature. The model yielded *R*^*2*^*X* = 0.799, *R*^*2*^*Y* = 0.999, and *Q*^*2*^*Y* = 0.965, and the corresponding 200-permutation test did not indicate obvious model overfitting. Nevertheless, because only three bottle-level fermentation replicates were included per treatment, the model and its discriminatory performance should be interpreted as exploratory.

Using the predefined primary criteria of VIP ≥ 1 and nominal *p* < 0.05, 168 differential volatile features were selected between FW and PW, including 72 with higher and 96 with lower normalised responses in FW (Fig. 6c). This comparison represents pomace-associated variation under the same fermentation condition of 20 °C. A more stringently screened and chemically curated subset of the FW-versus-PW differential features is provided in Table S2.

The FW-versus-CK comparison identified 145 differential features, including 109 with higher and 36 with lower normalised responses in FW (Fig. 6d). Because both treatments were prepared from juice without pomace, this comparison represents the most direct temperature-associated contrast between fermentation at 20 and 26 °C. The PW-versus-CK comparison identified 138 differential features, including 102 with higher and 36 with lower normalised responses in PW (Fig. 6e); however, this comparison combines differences in both fermentation temperature and substrate composition.

Features tentatively assigned to α-calacorene, isopentyl hexanoate, ethyl 2-octenoate, carvone, levomenthol, guaiacol, and methyl decanoate were among the labelled differential features in Fig. 6c–e. These tentative assignments do not establish that the corresponding compounds were key aroma contributors because they were not confirmed using authentic standards or experimentally determined retention indices, and quantitative concentrations, odour thresholds, and odour-activity values were unavailable.

Overall, fermentation temperature and pomace addition were associated with differences in the final volatile profiles. However, because a pomace-containing treatment fermented at 26 °C was not included, their independent effects cannot be fully separated. Moreover, the normalised GC–MS responses represent compositional variation rather than absolute concentrations. The present results therefore do not support ranking the treatments according to aroma intensity or quality.

### 3.4 Associations between microbial communities and non-volatile metabolite features

Spearman correlation analysis revealed stage-associated relationships between selected microbial genera and tentatively annotated non-volatile metabolite features in FW and PW fermented at 20 °C (Fig. 7a–c and e–g). During the early stage, *Paucibacter* was positively associated with features tentatively assigned to cis-11-methyl-2-dodecenoic acid, tryptophan–tyrosine, 11,20-dihydroxysugiol, dTDP-L-rhamnose, and an organic-acid-related derivative (Fig. 7a). These features were provisionally associated with fatty-acid-, peptide-, terpenoid-, nucleotide-sugar-, and organic-acid-related metabolism.

**Fig. 7:**
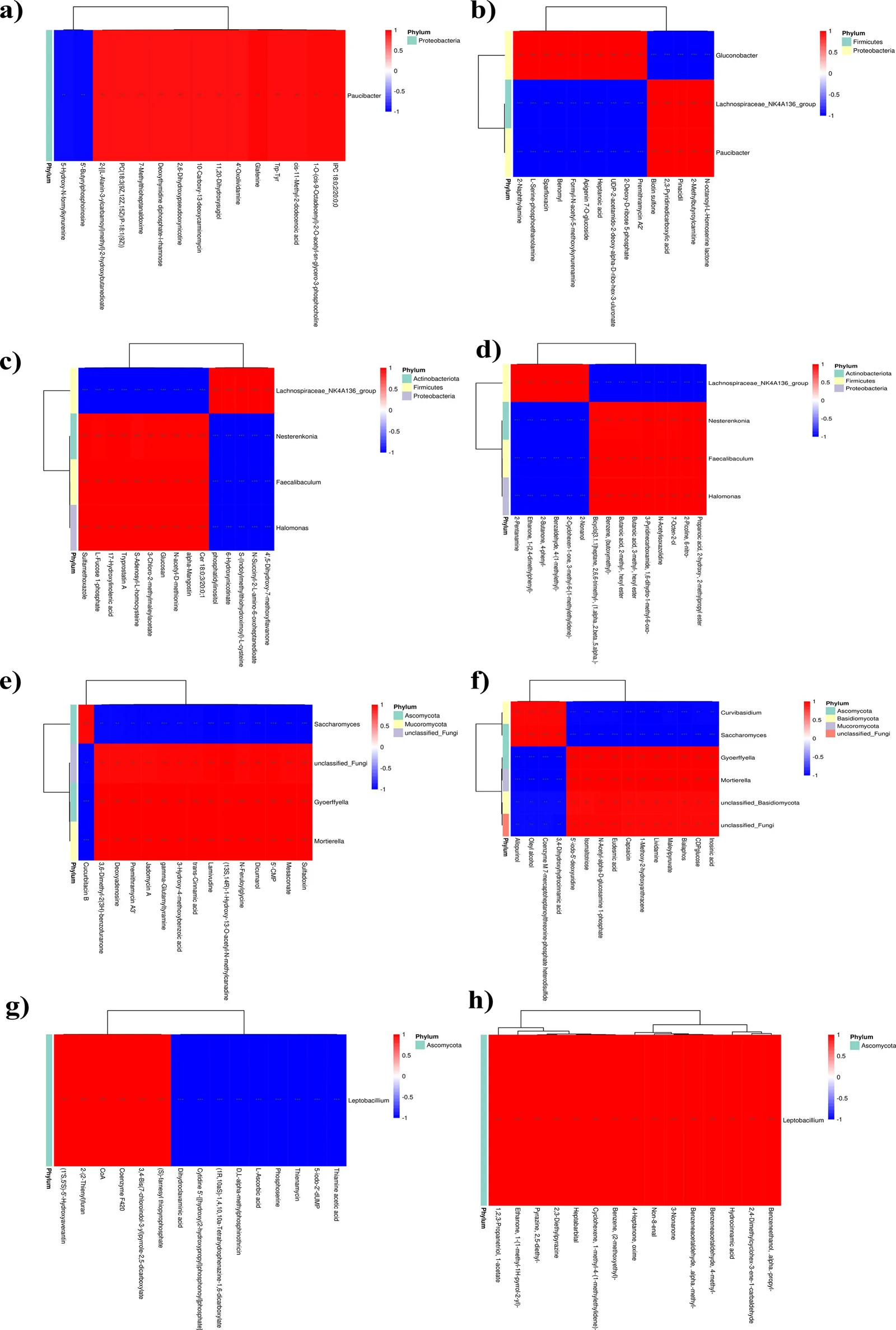
Exploratory associations between microbial genera and metabolite features in FW and PW fermented at 20 °C. Spearman correlation heatmaps between bacterial genera and putatively annotated non-volatile features at the early (a), middle (b), and late (c) stages; bacterial genera and differential volatile-feature records at the final stage (d); fungal genera and putatively annotated non-volatile features at the early (e), middle (f), and late (g) stages; and fungal genera and differential volatile-feature records at the final stage (h). Red and blue indicate positive and negative correlations, respectively. Only associations with |ρ| ≥ 0.8 and nominal *p* < 0.05 are shown. Each stage-specific analysis included six bottle-level fermentation samples (three FW and three PW replicates). Feature names indicate putative annotations based on database matching. (For interpretation of the references to colour in this figure legend, the reader is referred to the web version of this article.)

During the middle stage, *Gluconobacter* was positively associated with features tentatively assigned to 2-deoxy-d-ribose 5-phosphate, a UDP-linked carbohydrate derivative, heptanoic acid, and apigenin 7-*O*-glucoside (Fig. 7b). *Paucibacter* and the Lachnospiraceae_NK4A136_group showed association patterns that were generally opposite to those of *Gluconobacter* across the selected features. During the late stage, *Nesterenkonia*, *Faecalibaculum*, and *Halomonas* displayed similar associations with features related to amino-acid, lipid, and carbohydrate metabolism, whereas the Lachnospiraceae_NK4A136_group showed an opposing pattern (Fig. 7c).

Fungal–metabolite associations also differed among stages. During early fermentation, *Mortierella* was positively associated with features tentatively assigned to 5′-cytidine monophosphate, *N*-feruloylglycine, trans-cinnamic acid, 3-hydroxy-4-methoxybenzoic acid, and γ-glutamyltyramine (Fig. 7e). During the middle stage, *Curvibasidium* and *Saccharomyces* showed patterns opposite to those of *Mortierella* and *Gyoerffyella* across several selected features, including hydroxycinnamic-acid-, carbohydrate-, and fatty-alcohol-related signals (Fig. 7f). During the late stage, *Leptobacillium* was positively associated with several selected features, including one tentatively assigned to (*S*)-farnesyl thiopyrophosphate, whereas negative associations were observed with another subset of features (Fig. 7g).

These results indicate coordinated variation between microbial relative abundances and non-volatile metabolite signals but do not demonstrate direct microbial synthesis, degradation, or regulation. Given the small number of samples in each stage-specific analysis and the use of nominal *p* values without multiple-testing correction, the associations should be regarded as exploratory. Confirmation would require strain isolation, controlled fermentation, isotope tracing, or enzyme assays (Ding et al., 2024; Martins et al., 2024).

### 3.5 Associations of microbial genera with volatile features

Spearman correlation analysis identified associations between selected microbial genera and tentatively annotated volatile features in the final-stage FW and PW samples fermented at 20 °C (Fig. 7d and h). *Nesterenkonia* was positively associated with a feature tentatively assigned to 7-octen-2-ol (ρ = 0.988, nominal *p* = 2.02 × 10^−4^; Fig. 7d), whereas *Leptobacillium* was negatively associated with the same feature (ρ = −0.990, nominal *p* = 1.43 × 10^−4^; Fig. 7h). These opposing patterns indicate coordinated variation between microbial relative abundances and volatile-feature responses but do not establish microbial production, degradation, or regulation of 7-octen-2-ol.

*Leptobacillium* was also positively associated with features tentatively assigned to cyclohexene, 1-methyl-4-(1-methylethylidene)- (ρ = 0.999, nominal *p* = 8.32 × 10^−7^), hydrocinnamic acid (ρ = 0.997, nominal *p* = 1.40 × 10^−5^), and benzeneethanol, α-propyl- (ρ = 0.997, nominal *p* = 1.74 × 10^−5^). These results suggest concurrent changes in *Leptobacillium* abundance and selected terpenoid-, aromatic-acid-, and alcohol-related signals.

Microbial–volatile correlation analysis has similarly been used to screen candidate associations in fermented beverages (Liu, Chen, et al., 2024; Martins et al., 2024). However, the present volatile assignments were based on mass-spectral-library matching without confirmation using authentic standards or experimentally determined retention indices. Direct biosynthesis and sensory relevance therefore cannot be inferred from these associations.

Because the analysis included only six bottle-level fermentation samples and multiple genus–feature pairs were tested without multiple-testing correction, the reported *p* values are nominal and the results should be interpreted as exploratory. Validation through strain isolation, controlled fermentation, isotope tracing, quantitative analysis, and enzyme assays is required to establish causal microbial–volatile relationships.

## 4 Conclusion

This study characterised stage-associated changes in the microbial communities and chemical profiles of *Phyllanthus emblica* L. fruit wines produced from juice at 20 °C (FW), juice supplemented with pomace at 20 °C (PW), and juice at 26 °C (CK). Microbial composition differed among the treatment–stage combinations, with *Saccharomyces* representing a dominant fungal genus in several groups and *Gluconobacter* showing transient enrichment during PW fermentation. Untargeted LC–MS analysis of FW and PW yielded 2936 tentatively annotated non-volatile metabolite features. Forty annotated differential features were shared among the three stage-matched PW-versus-FW comparisons, suggesting pomace-associated differences across fermentation stages. HS-SPME–GC–MS analysis further revealed differences in the qualitative and proportional composition of the final volatile profiles. Exploratory correlations were observed between selected microbial genera, including *Nesterenkonia* and *Leptobacillium*, and non-volatile or volatile features; however, these associations do not demonstrate direct microbial metabolism or causality. Because DNA-based amplicon sequencing does not establish microbial viability or metabolic activity, the observed taxonomic patterns and microbial–metabolite associations should be interpreted cautiously. Moreover, the absence of a pomace-containing treatment fermented at 26 °C prevents complete separation of temperature and pomace effects. Factorial fermentation experiments, targeted quantification, viability- or activity-based microbial analyses, and sensory evaluation are therefore required to confirm the independent effects and underlying mechanisms.

## CRediT authorship contribution statement

**Tingmei Qi:** Writing – original draft, Software, Methodology, Data curation. **Xinyong Zhang:** Resources, Methodology, Investigation, Data curation. **Ni Liu:** Methodology, Data curation, Conceptualization. **Mingju Wan:** Validation, Investigation. **Yang Tian:** Resources, Conceptualization. **Qi Lin:** Methodology, Conceptualization. **Yuanyuan Bao:** Writing – review & editing, Project administration, Funding acquisition.

## Funding

This work was supported by the Joint Fund of the Yunnan Provincial Basic Research Program (Agriculture Field) under Grant No. 202401BD070001-048. The funding bodies had no role in the study design; sample collection; data analysis or interpretation; manuscript preparation; or the decision to submit the article for publication.

## Declaration of competing interest

The authors declare that they have no known competing financial interests or personal relationships that could have appeared to influence the work reported in this paper.

## Data Availability

The raw bacterial 16S rRNA gene and fungal ITS sequencing data have been deposited in the NCBI Sequence Read Archive under BioProject accession number PRJNA1503743. The processed non-volatile metabolomic and volatile-profile data supporting the findings of this study are provided in the article and its Supplementary Material. Additional raw data are available from the corresponding author upon reasonable request.
